# Effects of Ammonia Stress on Liver Tissue Structure, Enzyme Activities, and Metabolome of Juvenile Largemouth Bass *Micropterus salmoides*

**DOI:** 10.3390/metabo14120649

**Published:** 2024-11-21

**Authors:** Decheng Pu, Zhengxi Wang, Jishu Zheng, Peiyuan Li, Xiuli Wei, Dongsheng Li, Lihong Gao, Lin Zhou, Yu Wang

**Affiliations:** 1 Key Laboratory of Smart Agricultural Technology in the Southwest Mountains, Ministry of Agriculture and Rural Affairs (Co-Construction by Ministry and Province), Chongqing Academy of Agricultural Sciences, Chongqing 400715, China; 13036328893@163.com (Z.W.); imzhengjishu@163.com (J.Z.); lpy0616@126.com (P.L.); wei77703863@126.com (X.W.); dawson.lee@foxmail.com (D.L.); grantsan@163.com (L.G.);; 2 College of Fisheries, Southwest University, Chongqing 400715, China

**Keywords:** ammonia toxicity, oxidative stress, immune enzymes, tissue damage, metabolites

## Abstract

**Background:** Ammonia, a ubiquitous contaminant in aquatic ecosystems, poses multifaceted threats to fish species at elevated concentrations. **Methods:** In order to investigate the toxic effects of chronic ammonia stress on the liver of juvenile *Micropterus salmoides*, the present experiment was conducted to investigate the differences in changes in liver tissue structure, enzyme activities, and metabolomes after 28 days of ammonia exposure (0, 4, 8, and 16 mg/L). **Results:** The findings revealed that ammonia exposure induced significant oxidative stress in the liver, manifesting in decreased activities of antioxidant enzymes SOD and GSH-Px, elevated levels of GSH, GST, and MDA, and heightened activities of immune enzymes LZM, ALP, and ACP. An increase in ammonia concentration exacerbated liver tissue damage. Metabolome analysis further unveiled perturbations in liver metabolites of *Micropterus salmoides* exposed to ammonia, with Ala-His emerging as a potentially pivotal functional substance under chronic stress. Specifically, the 4 mg/L group responded to ammonia toxicity by augmenting GSH and L-Carnosine levels, the 8 mg/L group detoxified via upregulation of L-Glutamine, and the 16 mg/L group mitigated toxicity through the urea synthesis pathway. **Conclusions:** This research offers preliminary insights into the toxicological responses of *Micropterus salmoides* under chronic ammonia stress. It is suggested that the duration of ammonia concentration exceeding 4 mg/L in high-density aquaculture should not exceed 7 days.

## 1. Introduction

Ammonia represents a pivotal aquatic environmental impact factor in aquaculture, predominantly originating from the decomposition of organic matter and fish excreta in aquatic systems [1]. In aquaculture waters, ammonia primarily exists in the form of ionic ammonia (NH_4_^+^) and non-ionic ammonia (NH_3_). The NH_3_ can permeate through the gills and other organs of fish, exerting potent toxic effects that damage gills, the hepatopancreas, intestines, and other vital organs, ultimately leading to cell apoptosis and mortality [2,3,4,5]. Additionally, it disrupts the immune system balance and induces metabolic alterations [6,7,8,9,10]. The liver serves as a primary target for ammonia toxicity in fish and plays a crucial role in detoxification [11,12]. Research has demonstrated that ammonia causes liver tissue damage and oxidative stress, manifesting as nucleus deformation, vacuolization, and alterations in antioxidant enzyme activities in aquatic species such as large yellow croaker *Larimichthys crocea* [13], hybrid sturgeon *Acipenser baerii ♀ × Acipenser schrencki ♂* [14], salmonids *Salmo salar* [11], and Wuchang bream *Megalobrama amblycephala* [15]. Furthermore, ammonia also induces metabolic dysfunction in fish species including Nile tilapia *Oreochromis niloticus* [16], rainbow trout *Oncorhynchus mykiss* [17], and cuttlefish *Sepia pharaonis* [9]. Currently, the toxic effects of ammonia on fish livers are a focal area of research. However, there is a scarcity of studies examining the chronic toxic effects of long-term ammonia stress on fish.

*Micropterus salmoides*, a native species of the Mississippi River system in California, USA, is renowned for its rapid growth, palatable flesh, and substantial economic worth, making it a prominent aquaculture species in China with a production volume exceeding 800,000 tons in 2022 [18]. As high-density aquaculture techniques advance, the impacts of ammonia stress on *Micropterus salmoides* have garnered increasing attention. Current research primarily focuses on acute and short-term stress responses. Acute high ammonia stress has been shown to elicit oxidative stress in the liver [19], significantly alter the alpha and beta diversity of intestinal microbiota [20], damage liver and intestinal tissues, and suppress the expression of hsp70 mRNA in liver and gill tissues [21]. Short-term ammonia stress disrupts liver architecture, and affects serum alanine aminotransferase, aspartate aminotransferase, and alkaline phosphatase activities, leading to the upregulation of oxidative phosphorylation and downregulation of mitogen-activated protein kinase signaling pathways in the liver [22]. Furthermore, it affects ammonia excretion and reduces catabolism [23]. The cultivation of *Micropterus salmoides* is susceptible to various factors such as feeding practices, dissolved oxygen levels, and microbial balance, resulting in fluctuations in ammonia concentration in the water ranging from 0.24 mg/L to 5.3 mg/L [24]. This concentration range exceeds the safe level of ammonia for the F1 generation of the northern subspecies of *Micropterus salmoides* introduced, which is 2.51 mg/L, and approaches the safe concentration for domesticated *Micropterus salmoides* in China, which is 6.33 mg/L [22]. Therefore, the chronic presence of ammonia in aquaculture waters lends greater production significance to conducting studies on chronic stress. Currently, limited research exists on the liver tissue structure, antioxidant system, and metabolome of juvenile *Micropterus salmoides* under chronic ammonia stress. In this study, we examined changes in liver tissue structure, antioxidant enzymes, immune enzymes, and metabolome in juvenile *Micropterus salmoides* under chronic ammonia stress. This work aimed to analyze the toxicological effects of chronic ammonia stress and provide insights into healthy cultivation and water quality management of *Micropterus salmoides*.

## 2. Materials and Methods

### 2.1. Experimental Fish and Experimental Design

The juvenile *Micropterus salmoides* utilized in this experiment originated from the Chongqing Municipal Factory Agriculture Research and Development Center. Healthy individuals, possessing a body mass of 52.98 ± 2.63 g and a body length of 13.09 ± 0.27 cm, were randomly selected and placed in twelve culture ponds (each with a volume of 0.4 m^3^, diameter × height: 1 m × 0.5 m), for a 14-day acclimation period. During this time, fish were fed to the point of saturation two times a day, once in the morning and once in the evening, with a daily water exchange rate of 20% of the pond volume. Following acclimation, the ponds were randomly divided into one control group and three ammonia stress groups, with three replicates per concentration gradient, and each pond housed 100 experimental fish. The control group was maintained in aerated tap water. The treatment groups were exposed to ammonia concentrations of 4, 8, and 16 mg/L, respectively, based on the range observed in high-density cultures by the research team and referencing the 96-h LC_50_ of *Micropterus salmoides* [25]. Total ammonia concentration was monitored every 6 h using the “Determination of Ammonia Nitrogen in Water Quality-Gas Phase Molecular Absorption Spectrometry” (HJ 195-2023) method, and adjusted using the pre-prepared 10 g/L NH_4_Cl solution to maintain the desired concentrations for a total of 28 days. Throughout the experiment, dissolved oxygen was maintained at 8 ± 0.3 mg/L, water temperature at 26 ± 1 °C, pH at 7.0 ± 0.1, and feeding and water exchange rates remained consistent with the acclimation period.

### 2.2. Sample Collection

Samples were collected at 0, 3, 7, 14, and 28 days of the experiment, with a 24 h fasting period preceding each sampling. From each parallel group, four fish were randomly selected, and a total of 48 fish were sampled each time. Then they were anesthetized using 100 mg/L MS-222, and dissected on an ice tray to promptly excise liver tissues. A portion of the liver tissue was placed in a 10 mL centrifuge tube and stored at −80 °C for subsequent enzyme activity analysis. In addition, two liver tissues of *Micropterus salmoides* at 28 d were taken; one was put into 4% paraformaldehyde solution for staining of tissue sections, and the other was put into a 2 mL centrifuge tube, quick-frozen in liquid nitrogen for 15 min, and then stored in a refrigerator at −80 °C for metabolomics analysis.

### 2.3. Organizational Structure of the Liver

The liver tissues underwent fixation in 4% paraformaldehyde (PFA) for over 24 h, followed by dehydration through a graded series of ethanol concentrations (75%, 85%, 90%, 95%, and 100%). Subsequent to two hyalinization treatments with xylene, the tissues were embedded in paraffin wax. Five-micrometer slices were prepared, which were then deparaffinized using xylene and alcohol, followed by rinsing with distilled water. The slices were stained with hematoxylin-eosin (HE) [25]. After air-drying, the sections were sealed and analyzed using an Olympus Bx43 microscope (Olympus, Tokyo, Japan).

### 2.4. Enzyme Activity Assay

Liver samples were obtained from 10 mL centrifuge tubes and homogenized with saline in a 1:9 ratio. The homogenate was centrifuged at 9000 rpm for 10 min at 4 °C, and the resultant supernatant was used to assess the levels of antioxidant enzymes, including glutathione (GSH), superoxide dismutase (SOD), glutathione peroxidase (GSH-Px), glutathione-s-transferase (GST), and malondialdehyde (MDA). Additionally, immunoenzymatic activities of lysozyme (LZM), alkaline phosphatase (ALP), and acid phosphatase (ACP) were determined. The assays were conducted using kits from Nanjing Jiancheng Bioengineering Institute (Nanjing, China), following the manufacturer’s instructions meticulously.

### 2.5. Broadly Targeted Metabolomics

#### 2.5.1. Sample Preparation and Extraction

The sample stored at −80 °C was thawed on ice, and then ground with liquid nitrogen. A 400 μL solution of methanol and water (7:3, *v/v*), containing an internal standard, was added to 20 mg of the ground sample and shaken at 2500 rpm for 5 min. After being placed on ice for 15 min, the sample was centrifuged at 12,000 rpm for 10 min at 4 °C. A 300 μL aliquot of the supernatant was collected and stored at −20 °C for 30 min. Subsequently, the sample was centrifuged again at 12,000 rpm for 3 min at 4 °C. Finally, 200 μL aliquots of the supernatant were transferred for LC-MS analysis.

#### 2.5.2. T3 UPLC Conditions

The sample extracts were analyzed using an LC-ESI-MS/MS system (UPLC, ExionLC AD, https://sciex.com.cn/ (accessed on 7 August 2023); MS, QTRAP^®^ System, https://sciex.com/ (accessed on 7 August 2023)). The analytical conditions were as follows: UPLC: column, Waters ACQUITY UPLC HSS T3 C18 (1.8 µm, 2.1 mm × 100 mm); column temperature, 40 °C; flow rate, 0.4 mL/min; injection volume, 2 μL; solvent system, water (0.1% formic acid): acetonitrile (0.1% formic acid); gradient program, 95:5 *v/v* at 0 min, 10:90 *v/v* at 11.0 min, 10:90 *v/v* at 12.0 min, 95:5 *v/v* at 12.1 min, 95:5 *v/v* at 14.0 min.

#### 2.5.3. ESI-QTRAP-MS/MS

LIT and triple quadrupole (QQQ) scans were acquired on a triple quadrupole-linear ion trap mass spectrometer (QTRAP), QTRAP^®^ LC-MS/MS System (Waters, Milford, MA, USA), equipped with an ESI Turbo Ion-Spray interface, operating in positive and negative ion mode and controlled by Analyst 1.6.3 software (Sciex). The ESI source operation parameters were as follows: source temperature 500 °C; ion spray voltage (IS) 5500 V (positive), −4500 V (negative); ion source gas I (GSI), gas II (GSII), and curtain gas (CUR) were set at 55, 60, and 25.0 psi, respectively; the collision gas (CAD) was high. Instrument tuning and mass calibration were performed with 10 and 100 μmol/L polypropylene glycol solutions in QQQ and LIT modes, respectively. A specific set of MRM transitions was monitored for each period according to the metabolites eluted within this period.

Mass spectrometry data were processed using the software Analyst 1.6.3 for qualitative and quantitative mass spectrometry analysis of the metabolites of the samples based on local metabolic databases. The metabolome data were analyzed using Principal Component Analysis (PCA), Orthogonal Partial Least Squares-Discriminant Analysis (OPLS-DA), unsupervised hierarchical clustering, and volcano plots. Univariate analysis was employed to calculate the statistical significance (*p*-value) of each metabolite between the two groups through t-tests and to determine the fold change (FC) values between the groups. The metabolites with significant differences were further screened based on Variable Importance in Projection (VIP) obtained from the OPLS-DA model (biological replicates ≥ 3) combined with *p*-value/FDR (biological replicates ≥ 2) from univariate analysis. The identified metabolites were annotated using the KEGG database (https://www.kegg.jp/ (accessed on 10 September 2023)) and analyzed for heterometabolite metabolic pathway enrichment.

### 2.6. Statistics and Analysis

Experimental data were expressed as mean ± standard error (Mean ± S.E.). Data were analyzed by one-way ANOVA (One-way ANOVA) using SPSS 23 (IBM, Chicago, IL, USA) and multiple comparisons were performed using the Tukey HSD method. The significance level *p* was set at 0.05 and 0.01; when *p* ≤ 0.05, the difference was significant; when *p* ≤ 0.01, the difference was highly significant. Bar graphs were drawn by GraphPad Prism 9, and microstructure images were labeled using Adobe Photoshop 2022. Volcano plots, cluster heatmaps, and bubble plots for metabolomic analysis were drawn using R software (version 4.3.3).

## 3. Results

### 3.1. Effects of Ammonia on the Liver Tissue Structure of Micropterus salmoides

**Figure 1:**
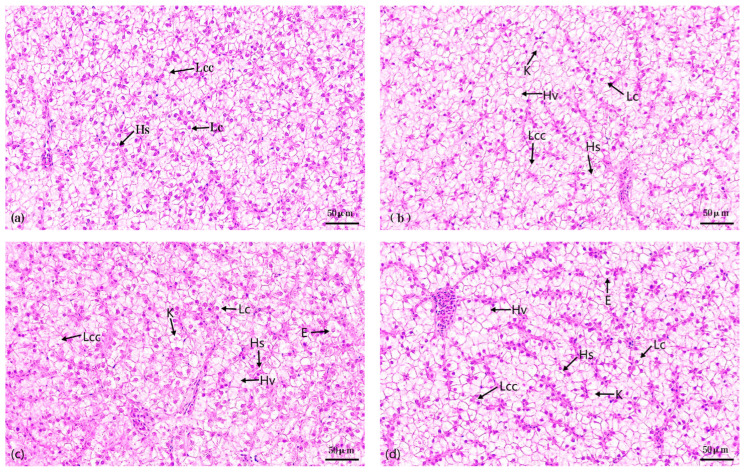
Effects of 28 d exposure to different ammonia concentrations on the liver tissue structure of *Micropterus salmoides.* Note: (**a**). control group; (**b**). 4 mg/L treatment group; (**c**). 8 mg/L treatment group; (**d**). 16 mg/L treatment group; Lc. hepatocytes; Hs. hepatic sinusoids; Lcc. hepatocyte cords; K. hepatocyte nucleolysis; Hv. hepatocyte vacuolation; E. hepatocyte enlargement.

A microscopic assessment of hepatic tissue architecture was conducted for both control and treatment groups at 28 d (Figure 1). In the control group, hepatocytes were densely arranged, exhibiting well-defined nuclear borders and relatively normal cellular structures. The hepatic sinusoids displayed normal morphology (Figure 1a). In contrast, the 4 mg/L treatment group revealed nuclear lysis, accompanied by cellular vacuolation (Figure 1b). The 8 and 16 mg/L treatment groups exhibited a more disrupted hepatocyte arrangement, with notable enlargement of hepatocytes. Additionally, there was an augmentation in nuclear lysis and cellular vacuolation, as well as marked dilatation of the hepatic sinusoids (Figure 1c,d).

### 3.2. Effect of Ammonia on Antioxidant Indices of Micropterus salmoides Liver

**Figure 2:**
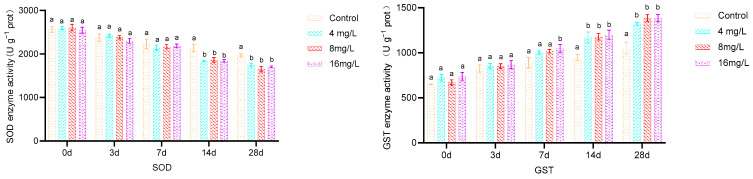

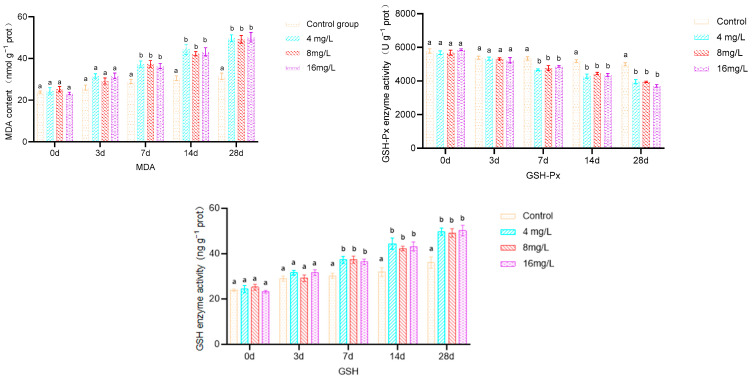
Effect of 28 d exposure to different ammonia concentrations on liver antioxidant enzyme activities of *Micropterus salmoides.* Note: Different letters indicate significant differences between groups with different ammonia concentrations at the same time (*p* < 0.05); same below.

As depicted in Figure 2, upon prolonged exposure to ammonia, the activities of SOD and GSH-Px exhibited a declining pattern in the 4, 8, and 16 mg/L treatment groups, whereas GSH content, GST activity, and MDA levels demonstrated an upward trend. No significant differences (*p* > 0.05) were observed in SOD, GSH-Px, GSH, GST, and MDA levels in the livers of all treatment groups compared to the control group at 3 d. SOD and GSH-Px activities were significantly reduced in all treatment groups compared to the control (*p* < 0.05) at 7 d, while GSH and MDA levels were significantly elevated (*p* < 0.05). GST activity in the 16 mg/L group was significantly higher than that in the 4 mg/L, 8 mg/L, and the control groups (*p* < 0.05). SOD and GSH-Px activities remained significantly lower (*p* < 0.05) at 14 and 28 d, while GSH, GST, and MDA levels were significantly higher (*p* < 0.05) in all treatment groups compared to the control. No significant differences were observed among treatment groups within the same time points (*p* > 0.05).

### 3.3. Effect of Ammonia on the Activity of Liver Immunoenzymes in Micropterus salmoides

**Figure 3:**
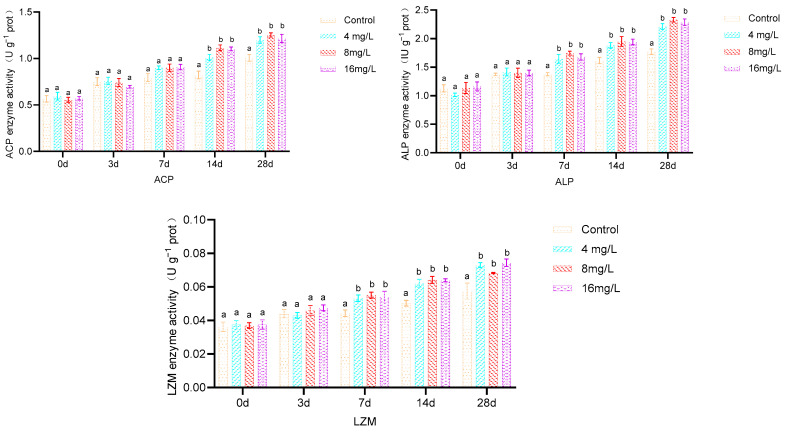
Effect of 28 d of exposure to different ammonia concentrations on liver immunoenzyme activities of Micropterus salmoides.

As depicted in Figure 3, the enzymatic activities of LZM, ALP, and ACP in the livers of *Micropterus salmoides* exhibited a general upward trend with prolonged exposure to ammonia concentrations of 4, 8, and 16 mg/L. No statistically significant differences (*p* > 0.05) were observed between the treatment groups and the control group in terms of LZM, ALP, and ACP levels at 3 d. However, the activities of these enzymes were significantly elevated in all treatment groups compared to the control group (*p* < 0.05) at 7, 14, and 28 d. No significant differences (*p* > 0.05) were discernible among the treatment groups during these respective comparison periods.

### 3.4. Effects of Ammonia on the Liver Metabolome of Micropterus salmoides

#### 3.4.1. Overall Changes in Metabolites

**Figure 4:**
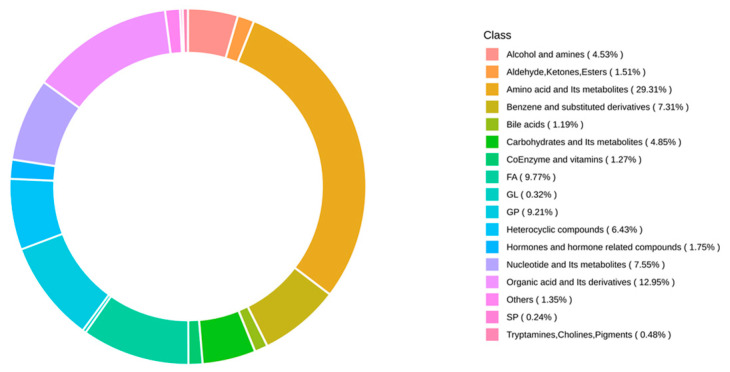
Ring diagram of metabolite class composition. Note: Each color represents a metabolite class, and the area of the color block indicates the percentage of that class that is represented.

**Figure 5:**
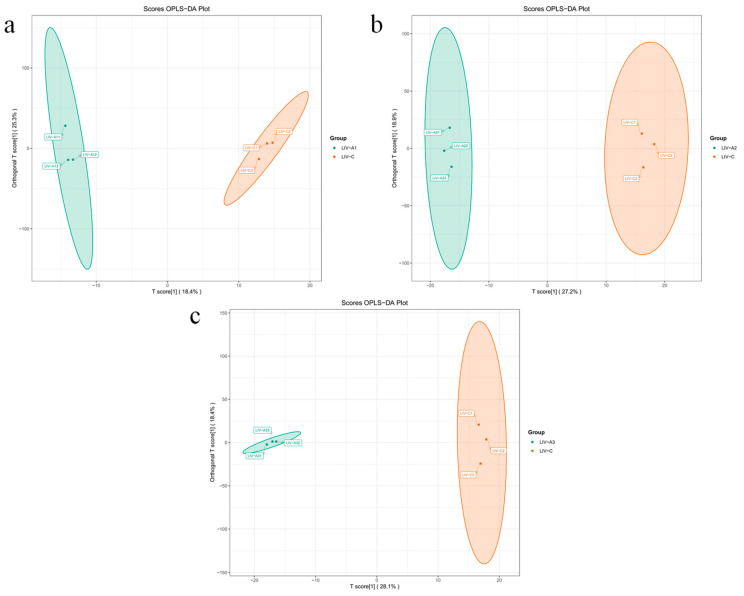
Map of OPLS-DA scores. (**a**), LIV−A1_vs._LIV−C; (**b**), LIV−A2_vs._LIV−C; (**c**), LIV−A3_vs._LIV−C. Note: the horizontal coordinate indicates the predicted principal component and the horizontal direction shows the gap between groups; the vertical coordinate indicates the orthogonal principal component and the vertical direction shows the gap within groups; and the percentage indicates the explanation rate of this component to the dataset. Each point in the graph represents a sample, samples in the same group are represented using the same color, and the Group is the subgroup.

An extensive metabolomic analysis using the UPLC-MS/MS was conducted on 12 *Micropterus salmoides* samples from four groups (control, 4, 8, and 16 mg/L treatments), which revealed 1259 metabolites. These metabolites encompass amino acid and their derivatives, organic acid and their derivatives, nucleotide and their metabolites, and benzene and substituted derivatives, among others (Figure 4). The groups were then categorized into three comparison sets: 4 mg/L vs. control, 8 mg/L vs. control, and 16 mg/L vs. control. Utilizing methods such as OPLS-DA, the metabolome data were rigorously analyzed. Figure 5 displays the visualization results of the OPLS-DA analysis for each comparative group, demonstrating a clear separation trend between the treatment and control groups, with statistically significant differences observed among them.

The control, 4 mg/L, 8 mg/L, and 16 mg/L treatment groups correspond to LIV−C, LIV−A1, LIV−A2, and LIV−A3, respectively; the same below.

#### 3.4.2. Differential Metabolite Screening

**Figure 6:**
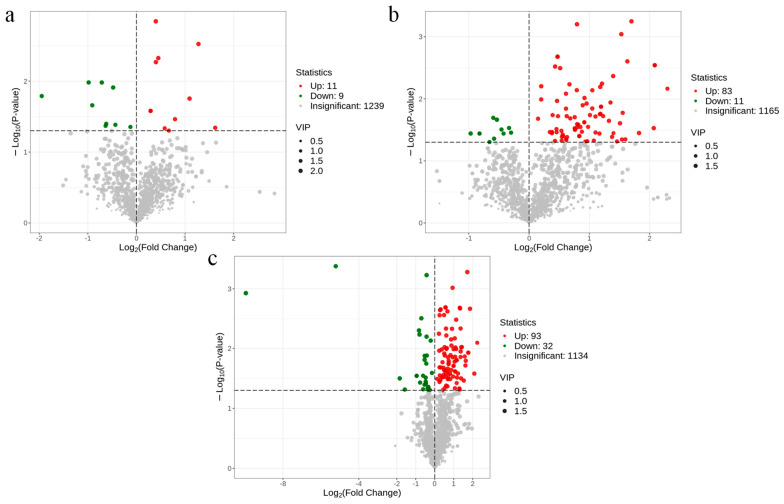
Volcano map of differential metabolites. (**a**), LIV−A1_vs._LIV−C; (**b**), LIV−A2_vs._LIV−C; (**c**), LIV−A3_vs._LIV−C. Note: each dot in the volcano diagram represents a metabolite, where green dots represent downregulated differential metabolites, red dots represent upregulated differential metabolites, and grey dots represent metabolites detected but with insignificant differences. The horizontal coordinate represents the logarithm of the multiplicity of the differences in the relative content of a metabolite between the two groups of samples (log2FC), and the greater the absolute value of the horizontal coordinate, the greater the difference of relative content of the substance between the two groups of samples. VIP + FC + *p*-value screening condition: the vertical coordinate indicates the significance level of difference (−log10*p*-value), and the size of the dot represents the VIP value.

**Figure 7:**
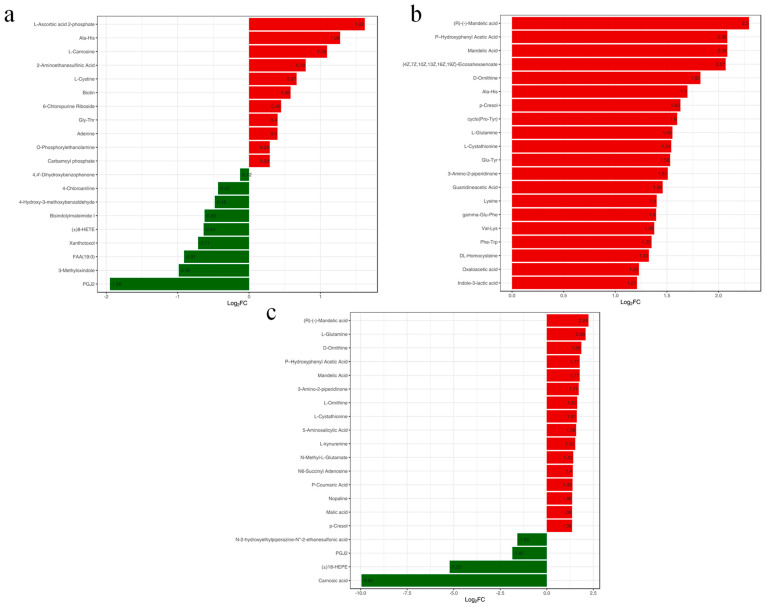
Variance multiplier bar graph. (**a**), LIV−A1_vs._LIV−C; (**b**), LIV−A2_vs._LIV−C; (**c**), LIV−A3_vs._LIV−C. Note: the horizontal coordinate is the log2FC of the differential metabolite, i.e., the value of the multiplicity of the difference of the differential metabolite taken logarithmically with 2 as the base, and the vertical coordinate is the differential metabolite. Red color represents upregulation of metabolite content and green color represents downregulation of metabolite content.

The differential metabolites were further screened using VIP (Variable Importance in Projection), FC (Fold Change), and *p*-value. Metabolites satisfying VIP > 1 and *p*-value < 0.05 were considered as differential metabolites. The volcano diagram (Figure 6) illustrates the metabolite alterations between each treatment group and the control, along with the count of significantly differing metabolites. Additionally, bar graphs depicting the multiplicity of differences for the top 20 metabolites in subgroup comparisons are presented in Figure 7. Our findings revealed that, in the 4 mg/L treatment group versus the control, 20 differential metabolites were identified, with 11 upregulated (e.g., L-Ascorbic acid 2-phosphate, Ala-His, L-Carnosine) and 9 downregulated (e.g., PGJ2, 3-Methyloxindole, FAA(19:0)) (Figure 6a). In the 8 mg/L treatment group, 94 differential metabolites were screened, comprising 83 upregulated and 11 downregulated metabolites; notably, all top 20 metabolites were upregulated (e.g., (R)-(−)-Mandelic acid, Mandelic acid, P-Hydroxyphenyl acetic acid) (Figure 6b). In the 16 mg/L treatment group, 125 differential metabolites were observed, with 93 upregulated (e.g., (R)-(−)-Mandelic acid, L-Glutamine, D-Ornithine) and 32 downregulated (e.g., Carnosic acid, (±)18-HEPE, PGJ2) (Figure 6c). These results underscore the changes in liver metabolites of *Micropterus salmoides* subsequent to exposure to varying concentrations of ammonia.

**Figure 8:**
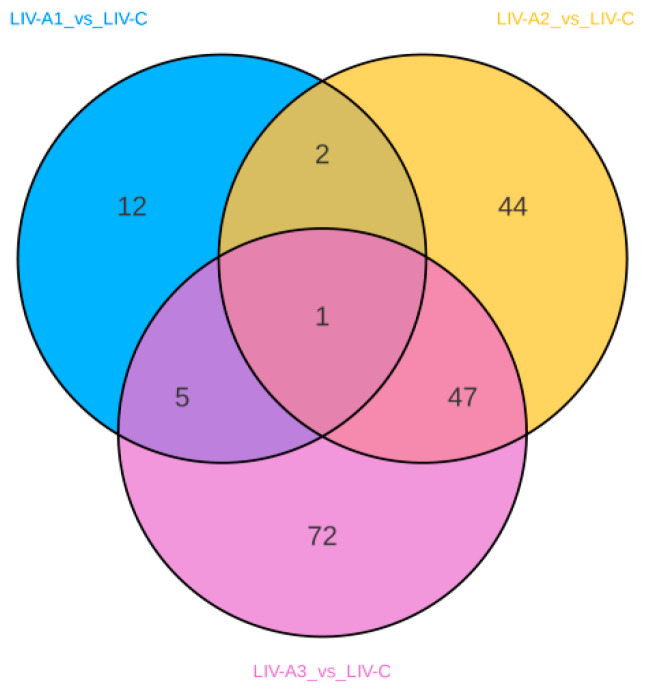
Venn diagram of differential metabolites in different comparison groups. Note: each circle in the figure represents a comparison group, the number of overlapping circles represents the number of differential metabolites common to the comparison groups, and the number with no overlap represents the number of differential metabolites specific to the comparison groups.

The relationship between the differential metabolites of the three comparison groups is shown in a Venn diagram (Figure 8). The results indicated that a shared differential metabolite existed among the three comparison groups. Different concentrations of ammonia stress all led to changes in the shared differential metabolite Ala-His in the liver of *Micropterus salmoides*.

#### 3.4.3. KEGG Metabolic Pathway of Differential Metabolites

**Figure 9:**
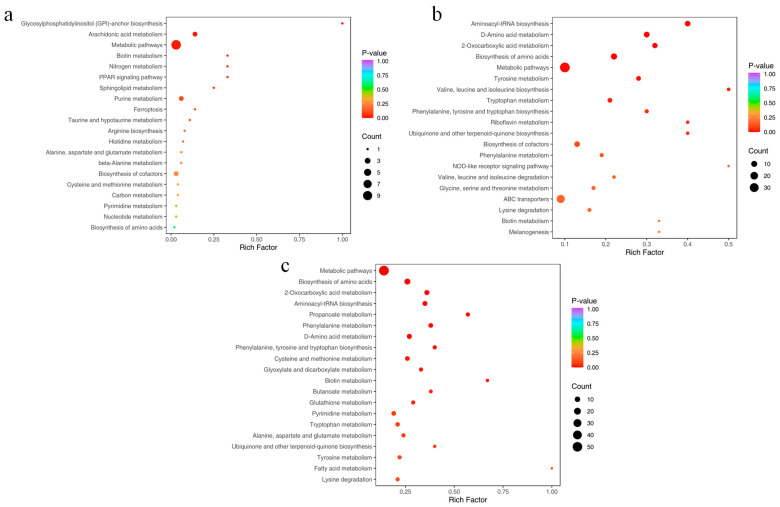
KEGG pathway of differential metabolites in the liver of *Micropterus salmoides* enriched in the top 20. (**a**), LIV−A1_vs._LIV−C; (**b**), LIV−A2_vs._LIV−C; (**c**), LIV−A3_vs._LIV−C. Note: the horizontal coordinate indicates the Rich Factor corresponding to each pathway. The vertical coordinate is the pathway name (sorted by *p*-value), and the color of the dots reflects the size of the *p*-value; the redder the more significant the enrichment. The size of the dots represents the number of different metabolites enriched.

**Figure 10:**
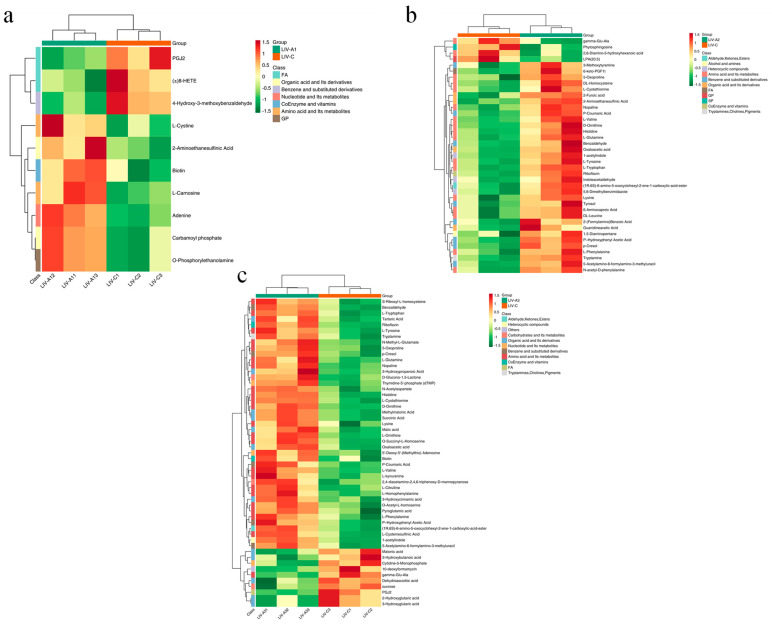
Heatmap of differential metabolite clustering of the KEGG pathway. (**a**), LIV−A1_vs._LIV−C; (**b**), LIV−A2_vs._LIV−C; (**c**), LIV−A3_vs._LIV−C. Note: the horizontal coordinate is the sample name. The vertical coordinate is the differential metabolite, different colors are the colors filled with different values obtained after the standardized treatment of different relative contents (red represents high content, green represents low content). The comment bar above the heat map corresponds to the grouping of the samples (Group). The dendrogram on the left side of the heat map represents the results of the hierarchical clustering of the differential metabolites. The comment bar on the right side of the clustering tree corresponds to the substance class (Class), and different colors represent different substance classes. The annotation bars on the right side of the clustering tree correspond to the first class of substances, and different colors represent different substance classes.

All differential metabolites in the three comparison groups were matched against the KEGG database to obtain information on the pathways in which the metabolites were involved. Subsequent enrichment and analysis of annotated results identified the top 20 pathways based on *p*-value rankings, presented in ascending order in Figure 9. The findings revealed that, in comparison to the control group, the 4 mg/L treatment group primarily exhibited enrichment in metabolic pathways, arachidonic acid metabolism, purine metabolism, biosynthesis of cofactors, and glycosylphosphatidylinositol (GPI)-anchor biosynthesis (Figure 9a). The 8 mg/L treatment group sequentially showed enrichment in metabolic pathways, ABC transporters, biosynthesis of amino acids, aminoacyl-tRNA biosynthesis, and D-amino acid metabolism (Figure 9b). The 16 mg/L treatment group demonstrated enrichment in metabolic pathways, biosynthesis of amino acids, D-amino acid metabolism, 2-oxocarboxylic acid metabolism, and aminoacyl-tRNA biosynthesis (Figure 9c). These metabolic pathways were intricately linked to the hepatic metabolic profiles of *Micropterus salmoides* under ammonia stress. Specifically, the pathways in the 4 mg/L treatment group were significantly associated with FA and organic acid and their derivatives (Figure 10a). The pathways in the 8 and 16 mg/L treatment groups were predominantly correlated with amino acid and their metabolites, as well as organic acid and their derivatives (Figure 10b,c).

## 4. Discussion

### 4.1. Ammonia Stress Causes Liver Tissue Damage

The liver is an important organ for metabolism and detoxification in fish. The toxicity of chemicals is usually first seen in the liver, and ammonia stress can lead to structural damage to the liver in fish [26]. In the present study, following a 28 d exposure period, ammonia concentrations of 4 mg/L, 8 mg/L, and 16 mg/L all induced significant pathological alterations in the liver tissue of juvenile *Micropterus salmoides*. These changes, including hepatocyte disarray, swelling, and vacuolization, were comparable to those observed in studies involving 48 h acute and 7 day subchronic exposures to high ammonia concentrations [19,21]. The present study showed that the impact of various ammonia concentrations on liver tissue structure was differential. Specifically, 4 mg/L ammonia stress primarily led to nucleolysis and cellular hollowing. As the ammonia concentration increased to 8 and 16 mg/L, there was a notable augmentation in hepatocellular damage, nucleolysis, and cellular vacuolization, accompanied by hepatocyte enlargement, disorganized cellular arrangement, and dilatation of hepatic blood sinusoids. These results were consistent with those reported in studies involving Taiwan loach *Paramisgurnus dabryanus* ssp. Taiwan [27], *Verasper variegatus* [28], and *Megalobrama amblycephala* [4]. Our research indicated that liver tissues were susceptible to damage under long-term stress at an ammonia concentration of 4 mg/L. The damage was exacerbated by further increases in ammonia concentration.

### 4.2. Ammonia Exposure Induces Oxidative Stress in the Liver

Oxidative stress is a pivotal mechanism underlying toxicity in aquatic animals, and elevated ammonia concentrations in aquatic environments can provoke substantial generation of reactive oxygen species (ROS) in fish livers [26,29]. The SOD, which catalyzes the conversion of superoxide anion (O_2-_) to oxygen (O_2_) and hydrogen peroxide (H_2_O_2_), plays a crucial role in mitigating oxidative stress in organisms [30]. Additionally, GSH-Px, a selenium-dependent antioxidant enzyme located in the cytoplasm, catalyzes the reduction of hydrogen peroxide (H_2_O_2_) and lipid peroxides to water and lipid alcohols, thereby mitigating toxicity [31]. In the present study, ammonia stress resulted in a decline in SOD and GSH-Px activities in the liver of *Micropterus salmoides*. Specifically, GSH-Px activity was significantly reduced compared to the control group from 7 d onwards, while SOD activity decreased significantly from 14 d onwards in the treatment groups. These findings suggest that liver tissues consumed SOD and GSH-Px to counteract the oxidative stress induced by ammonia, aligning with previous observations in yellow catfish *Pelteobagrus fulvidraco* [32].

GST and GSH are vital antioxidant enzymes in hepatic tissues. GST catalyzes the conjugation of GSH with various electronegative compounds, facilitating their elimination and metabolism, thereby reducing oxidative stress-induced damage. GSH scavenges ROS, mitigating intracellular oxidative damage [33,34]. Previous studies have shown that ammonia stress elevates hepatic GST and GSH activities in Yellow River carp [35], increases GST content in Nile tilapia and Brazilian flounder *Paralichthys orbignyanus* livers [36,37], and boosts hepatic GSH activities in *Micropterus salmoides* and green carp [15,38]. Consistent with these findings, our study revealed that ammonia stress induced a significant increase in GST and GSH activities in *Micropterus salmoides*, starting from 7 d in all treatment groups. This suggests that with increasing ammonia stress, ROS stimulate antioxidant defense [26].

MDA is a marker of lipid peroxidation; levels of MDA in the liver are significantly elevated in fish when exposed to pollutants, reflecting lipid peroxidation and potential cellular damage [39,40]. The results of this study showed that liver MDA content increased under continuous stress from 4, 8, and 16 mg/L ammonia stress, with significant elevations compared to the control group starting from 7 d. This suggests that continuous ammonia stress induces oxidative stress in *Micropterus salmoides* livers, leading to MDA overproduction due to free radicals. To prevent excessive liver damage, antioxidant enzymes such as SOD, GSH-Px, GST, and GSH were activated. However, histological analysis of liver tissues confirmed cellular damage as early as 7 d (Figure 1).

### 4.3. Ammonia Exposure Affects Liver Immunity

LZM serves as a pivotal defensive agent in the innate immune system of fish and stands as a standard biomarker for assessing health and immune competence [41,42]. Prior research has demonstrated that high concentrations of acute ammonia stimulate *Micropterus salmoides* to synthesize or secrete LZM, thereby augmenting its activity and fortifying nonspecific immunity against ammonia toxicity [20]. Consistent with previous findings, our study revealed that all treatment groups elicited an elevation in hepatic LZM activity in *Micropterus salmoides*, with a significant increase observed from 7 d onwards compared to the control group.

ACP is a lysosomal enzyme integral to cellular autolysis and phagocytosis, and AKP is a membrane-associated enzyme with dephosphorylation capabilities [43,44]. They play crucial roles within the immune system. It was found that ammonia stress led to a significant increase in ACP and AKP activities in the liver cells of Nile tilapia to enhance their metabolism and detoxification [45]. In addition, ammonia stress has induced a marked increase in ACP and AKP activities in hybrid grouper *♀ Epinephelus fuscoguttatus* × *♂ E. lanceolatus* [46] and augmented ACP activity in hibiscus carp Furong *crucian carp*, thereby activating its non-specific immune system [47]. In the present study, all treatment groups induced enhanced hepatic ACP and AKP activities in *Micropterus salmoides*, and were significantly higher than the control group on day 7. The results indicated that the enhancement of LZM, ACP, and AKP activities activated the non-specific immune system of *Micropterus salmoides* under continuous ammonia stress at 4, 8, and 16 mg/L. However, the structural damage to the hepatocytes caused by prolonged ammonia stress led to the elevation of ALP activity [47].

### 4.4. Ammonia Exposure Alters the Hepatic Metabolic Profile

#### 4.4.1. Metabolism of Small Peptides

Small peptides are peptides formed by no more than 20 amino acids through peptide bonds [48]. During the digestive process in animals, the majority of proteins, under the enzymatic action within the gastrointestinal tract, are hydrolyzed into small peptides comprising two to three amino acid residues. These peptides are absorbed intact into the circulatory system and subsequently utilized by various tissues [49]. As biologically active compounds, small peptides possess the capacity to bind to specific receptors and elicit a range of biological effects, including the regulation of growth and development, augmentation of immune responses, metabolic modulation, and antimicrobial activity, through the modulation of cellular signaling pathways [50]. For example, Ala-His, Leu-Gly, and Pro-Gly-Pro peptides showed high protection against enzymatic and histological changes in the livers of diabetic mice [51]. In the protein hydrolysates of cod *Gadus morhua*, peptides containing His and Tyr, with a molecular weight below 600 Da, exhibit potent free radical scavenging activity [52].

In this study, the small peptide Ala-His, a dipeptide consisting of alanine and histidine, was identified as a differential metabolite that was upregulated and enriched in ABC transporters. Proteins belonging to the ABC transporter gene family harness the energy derived from ATP hydrolysis to actively transport a diverse array of substrates across lipid membranes. These proteins play pivotal roles in glucose and lipid metabolism, bile acid synthesis, and detoxification mechanisms within the liver [53,54]. Considering the alterations observed in hepatic antioxidant and immune enzyme activities, we infer that Ala-His exerts an antioxidant effect by scavenging free radicals in the liver, thereby mitigating hepatocyte damage. Furthermore, it enhances liver metabolic function and confers resistance to ammonia-induced toxic damage by enriching and upregulating the metabolic pathways associated with ABC transporters. The specific physiological functions of the small peptide Ala-His in the liver of *Micropterus salmoides* need to be further investigated. However, combining the common physiological functions of small peptides and the potential role of antioxidant peptides, the specific mechanism of Ala-His in the liver was revealed. It may provide new ideas for the prevention and treatment of liver injury caused by ammonia toxicity in *Micropterus salmoides*.

#### 4.4.2. Fatty Acid Metabolism

The KEGG pathway enrichment analysis revealed that the metabolite PGJ2 was significantly downregulated in the 4 and 16 mg/L treatment groups, and the metabolite 6-keto-PGF1α was significantly (*p* < 0.05) downregulated in the 8 mg/L treatment group. These differential metabolites were predominantly enriched within the arachidonic acid (ARA) metabolism pathway, with a particular enrichment observed in the 4 mg/L treatment groups. ARA is an omega-6 polyunsaturated fatty acid found in the phospholipids of biological cell membranes and lipid droplets of immune cells. Upon exposure to diverse stimuli, ARA is liberated from cellular membrane phospholipids via the catalytic action of enzymes such as phospholipase A2, phospholipase C, and phospholipase D. This liberation initiates a cascade of enzymatic reactions, ultimately converting ARA into biologically active metabolites, including thromboxane A2 (TXA2) and prostaglandins PGE2, PGI2, PGD2, and PGF2α. These metabolites exert pivotal roles in modulating inflammatory responses, immune regulation, facilitating tissue regeneration, and enhancing cellular proliferation [55,56]. PGJ2, an active product of arachidonic acid metabolism, is an endogenous inflammatory mediator derived from the non-enzymatic dehydration of PGD2, which further converts to Δ12-PGJ2 and 15-deoxy-Δ12,14-PGJ2 (15d-PGJ2) [57]. 15d-PGJ2 functions as an oxidative phosphatidylcholine proliferator-activated receptor gamma (PPARγ) ligand, exhibiting either anti-inflammatory or pro-inflammatory effects in cellular contexts and promoting fibroblast proliferation [58,59,60].

Furthermore, 6-keto-PGF1α, the primary metabolite of PGI2, has been demonstrated to regulate tissue inflammation, with increased levels typically observed in inflamed tissues [61,62,63,64]. In the present study, ammonia stress induced tissue damage in the liver of *Micropterus salmoides*, eliciting an inflammatory response and a significant downregulation of inflammatory endogenous PGJ2 and 6-keto-PGF1α within hepatic arachidonic acid metabolism. This downregulation may represent an adaptive metabolic regulatory response aimed at protecting against tissue damage. These findings suggest that ammonia stress elicits alterations in fatty acid metabolism within the liver of *Micropterus salmoides*.

#### 4.4.3. Amino Acid Metabolism

Within a certain range, fish usually alleviate ammonia toxicity by increasing the total free amino acid level and synthesizing glutamine and urea [65,66]. Metabolome analysis revealed that in the 8 mg/L treatment group, amino acids such as L-Phenylalanine, L-Valine, Histidine, L-Glutamine, Lysine, DL-Leucine, L-Tyrosine, and L-Tryptophan were significantly upregulated and primarily enriched in pathways involving ABC transporters, amino acid biosynthesis, aminoacyl-tRNA biosynthesis, and D-amino acid metabolism. Glutamine synthetase (GS) catalyzes glutamine synthesis, a crucial pathway for ammonia detoxification [67,68]. The marble goby *Oxyeleotris marmoratus* enhances glutamine content through the activation of hepatic glutamine synthetase, thereby reducing ammonia toxicity [69]. Thus, the significant upregulation of L-Glutamine in the 8 mg/L group represents a metabolic detoxification response.

In the 16 mg/L treatment group, amino acids including Lysine, L-Ornithine, L-Phenylalanine, L-Tryptophan, L-Tyrosine, L-Homophenylalanine, and L-Valine were significantly upregulated, and primarily enriched in pathways related to D-amino acid metabolism, ABC transporters, glutathione metabolism, and amino acid biosynthesis. The urea cycle, crucial for ammonia detoxification, involves the metabolism of arginine and ornithine [16]. In hepatic cells, ornithine is catalyzed by ornithine transcarbamylase (OTC) to produce citrulline, which then migrates to the cytoplasm to react with aspartate, generating arginine. The arginine is subsequently hydrolyzed by arginase (ARG) into urea and ornithine, with the ornithine once again participating in the synthesis of citrulline, thus sustaining the continuous production of urea [70,71]. Studies have shown that *Sepia pharaonis* and air-breathing magur catfish *Clarias magur* detoxify ammonia primarily through the urea cycle [9,72]. The significant upregulation of L-Ornithine in the 16 mg/L group may be associated with the ornithine–urea cycle to alleviate ammonia toxicity. When the urea cycle is inhibited or disturbed, *Pelteobagrus fulvidraco* responds to ammonia toxicity by increasing glutamine synthesis [73,74]. In contrast, the 8 mg/L group detoxified solely through the upregulation of L-Glutamine, with no detectable urea cycle detoxification pathway, potentially due to the inhibition of the hepatic urea cycle. These results are similar to those observed in *Micropterus salmoides* under short-term stress at a 13 mg/L concentration [23].

In the 4 mg/L treatment group, L-Cystine and L-Carnosine were significantly upregulated and primarily enriched in pathways involving ABC transporters, cysteine and methionine metabolism, ferroptosis, histidine metabolism, and beta-alanine metabolism. L-Cystine is taken up by cells and reduced to L-Cysteine, which is subsequently released outside the cell to maintain the L-Cysteine concentration [75]. Glutamate–cysteine ligase (GCL) catalyzes the production of glutathione (GSH) from L-Cysteine, which exhibits diverse functions such as detoxification, antioxidant defense, and the regulation of cell proliferation [76]. The increased hepatic GSH content in the 4 mg/L group suggests that the upregulation of L-Cystine promoted GSH synthesis under ammonia stress. L-Carnosine is a dipeptide composed of β-Alanine and L-Histidine, which have been demonstrated to possesses antioxidant, anti-inflammatory, and anti-fibrotic activities [77,78]. The regulation observed in *Micropterus salmoides* in the 4 mg/L group suggests that they cope with ammonia-induced liver damage by increasing GSH and L-Carnosine levels but lack the detoxification pathway for synthesizing glutamine and urea. Scleractinian fish possess ornithine–urea cycle (OUC) genes [79], and high ammonia stress induces OUC genes in *Clarias magur* hepatic and non-hepatic tissues [73]. However, only the 16 mg/L group mitigated ammonia toxicity through the urea synthesis pathway, potentially due to the induction of hepatic OUC genes in *Micropterus salmoides*. These findings indicate that ammonia stress disrupts amino acid metabolism in the liver of *Micropterus salmoides* and that it exhibits different metabolic detoxification pathways under varying ammonia concentrations.

## 5. Conclusions

The present study demonstrates that oxidative stress manifests in the livers of *Micropterus salmoides* exposed to ammonia stress concentrations of 4, 8, and 16 mg/L. Significant alterations in the activities of antioxidant and immune enzymes were observed, accompanied by tissue damage characterized by hepatocyte vacuolation. Furthermore, metabolomic analysis results indicate that ammonia stress induces changes in fatty acid and amino acid metabolism within the livers of *Micropterus salmoides*. The fish respond to varying concentrations of ammonia toxicity through various pathways, including glutamine and urea synthesis, as well as the enhancement of antioxidant defenses. These findings suggest that the management of ammonia should be taken seriously in aquaculture operations. It is recommended that the duration of ammonia concentrations exceeding 4 mg/L in high-density aquaculture systems should not exceed 7 days to mitigate the risk of chronic toxicity. Future research can delve further into the mechanisms and efficacy of ammonia-decomposing substances in treating ammonia toxicity, aiming to provide a reliable solution for mitigating ammonia poisoning in production processes.

## Data Availability

The data presented in this study are available on request from the corresponding author.
